# Probiotics, Prebiotics, and Synbiotics: Gut and Beyond

**DOI:** 10.1155/2012/872716

**Published:** 2012-09-19

**Authors:** Usha Vyas, Natarajan Ranganathan

**Affiliations:** Kibow Biotech Inc., Newtown Business Center, 4781 West Chester Pike, Newtown Square, PA 19073, USA

## Abstract

The human intestinal tract has been colonized by thousands of species of bacteria during the coevolution of man and microbes. Gut-borne microbes outnumber the total number of body tissue cells by a factor of ten. Recent metagenomic analysis of the human gut microbiota has revealed the presence of some 3.3 million genes, as compared to the mere 23 thousand genes present in the cells of the tissues in the entire human body. Evidence for various beneficial roles of the intestinal microbiota in human health and disease is expanding rapidly. Perturbation of the intestinal microbiota may lead to chronic diseases such as autoimmune diseases, colon cancers, gastric ulcers, cardiovascular disease, functional bowel diseases, and obesity. Restoration of the gut microbiota may be difficult to accomplish, but the use of probiotics has led to promising results in a large number of well-designed (clinical) studies. Microbiomics has spurred a dramatic increase in scientific, industrial, and public interest in probiotics and prebiotics as possible agents for gut microbiota management and control. Genomics and bioinformatics tools may allow us to establish mechanistic relationships among gut microbiota, health status, and the effects of drugs in the individual. This will hopefully provide perspectives for personalized gut microbiota management.

## 1. Introduction

Bacteria, unicellular eukaryotes, and other organisms inhabit the human body in large numbers. The human gut is dominated by several bacterial phyla including *Bacteroidetes*, *Firmicutes*, and *Actinobacteria*. The term “microbiota,” “microflora,” or “normal flora” is used to designate this vast host of microbes which coexist with the host [1–3]. 

It is estimated that the human microbiota contains as many as 10^14^ bacterial cells, a number that is 10 times greater than the number of human cells present in our bodies [4–6]. Virtually every surface of the human body starting from the skin surface to the genitourinary tract, oral cavity, respiratory tract, ear, and the gastrointestinal tract is colonized heavily by various species of bacteria [3, 7–9]. By far, the most heavily colonized organ is the gastrointestinal tract (GIT) which houses a huge microbial ecosystem; the colon alone is estimated to contain over 70% of all the microbes in the human body [4, 6]. 

The gut microbiota or microflora has a crucial role in human health and disease. The GIT is comprised of the entire digestive system from the stomach to the anus. The colon or the large intestine is the organ which is the preferred site for bacterial colonization. The GIT is also rich in many molecules which can be used as nutrients by microbes. Hence the GIT has the potential to be heavily colonized by various bacteria both harmful and beneficial. The mucosa of the gastrointestinal tract is continuously exposed to an environment that is rich in foreign substances, such as food particles and antigens of microbial origin. Particular changes in the intestinal ecosystem might contribute to the development of certain illness. There is therefore a need for an exhaustive review on the functions of the gut microbiota, occurrence of gut dysbiosis (alteration or imbalance of the microflora), how these intestinal bacteria trigger development of disease once the normal flora of a healthy individual is imbalanced, exploiting this intricate and interwoven ecosystem for understanding human health, development of biotherapeutics, and future perspectives. 

The imbalanced gut bacteria have been studied in diseases such as inflammatory bowel disease, antibiotic-associated diarrhea, colon cancer, hypercholesterolemia, and others. Lactic acid bacteria, belonging to the genus *Lactobacillus* and *Bifidobacterium*, have been shown to positively influence health. Hence, re-establishing the balance by using these bacteria (termed “probiotics”) for disease treatment and prevention should prove advantageous. Probiotics along with prebiotics and synbiotics have been used and studied in various disease areas. Several studies have indicated that an altered gut microbiota is associated with several diseases that are particularly prevalent in the 21st century. 

N. Williams [10] has previously reviewed the pharmacology, uses, dosage, safety, drug interactions, and contraindications of probiotics. The first part of this updated and current review will give an overview of the gut microbiota and its main characteristics and describe the major factors that could modulate gut microbiota composition. The second part describes various new diseases and reports on studies in which probiotics, prebiotics, and synbiotics have been used. The virtues of probiotics are already well recognized for general gut health, antibiotic-associated diarrhea, and immunity. Application of these areas will not be examined. The last part of the review focuses on future potential applications of probiotics, prebiotics and synbiotics in new emerging areas of studies like autism and gut-brain connection. Finally, the paper will conclude with a discussion on the future of this field. A short review article of this length cannot do full justice to this field. A broad overview, composed of excerpts taken from various publications including review articles, is presented here. For each of the important areas, we have included references to review articles for readers wishing to delve and analyze more deeply. 

## 2. The Gut Microbiota and Functions

**Figure 1:**
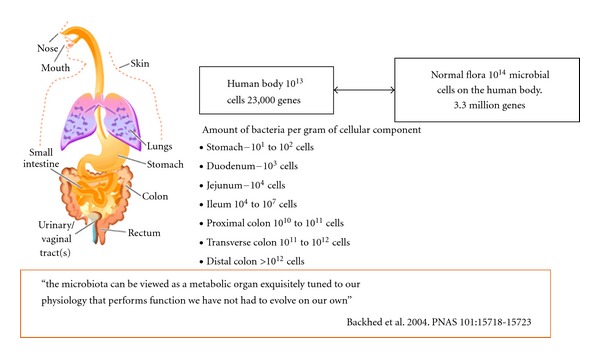
The Human Body and number of bacteria present in the total microflora.

A newborn baby has a sterile gut that is colonized by bacteria from the mother and from the baby's surroundings or environment [11]. An adult human has 10 times more bacterial cells on, and in, the entire body as compared to the total human cells (Figure 1). The human microbiome is highly complex and diverse. Its composition and number varies from the nose and mouth to the distal colon and rectum. The composition and complexity of the gut microbiota changes when the baby is weaned to solid foods. Dietary changes in adulthood are also greatly responsible for the composition of gut microbiota. Development of 16S ribosomal RNA (rRNA) gene-sequence-based metagenomic methods has led to major advances in defining the total microbial population of the gut [12]. This technique has been used to show that 90% of the bacteria belong to two phyla, namely, the Bacteroidetes and Firmicutes [13]. 

The gut microbiota plays an important role in the maintenance of health. These are summarized below.

### 2.1. Structure and Histological Function

The intestinal structure and function is ensured by the microbiota present within. The intestinal mucus layer is a balance of mucin secretion and degradation. This mucin layer creates an obstacle to proinflammatory compounds and uptake of antigens [14]. Evidence indicates that butyrate induces secretion of mucin, antimicrobial peptides, and other factors. This reinforces the defense barrier in the colon [15].

**Figure 2:**
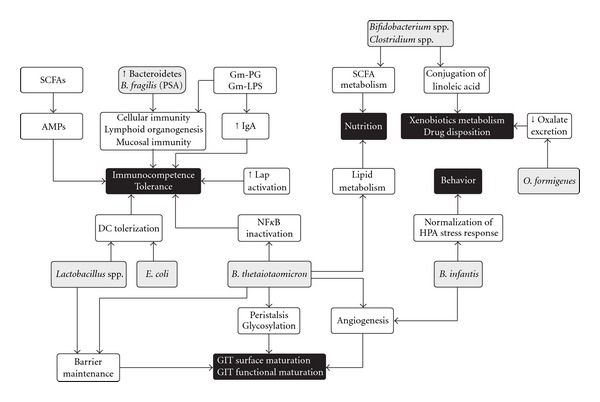
The complex web of gut microbiota contributions to host physiology. Different gut microflora components can affect many aspects of normal host development, while the microbiota as a whole often exhibits functional redundancy. Members of the microbiota are shown in gray, with their components or products of their metabolism. Their effects on the host at the cellular or organ level are shown in white. Black ellipses represent the affected host phenotypes. Only some examples of microbial members/components contributing to any given phenotype are shown. AMP: antimicrobial peptides; DC: dendritic cells; Gm−: gram negative; HPA: hypothalamus-pituitary adrenal; Iap: intestinal alkaline phosphatase; PG: peptidoglycan; PSA: polysaccharide A. Extracted from: Phys Rev 2010 Sekirov et al.

Secondly, the gut microflora has a role in the development of cell and tissue. Butyrate, a short chain fatty acid that is secreted by these colonic microbes, regulates cell growth and differentiation, inhibits transformation of cell growth and helps in reverting the cells from a neoplastic to a nonneoplastic phenotype [16]. The development of the microvasculature of the intestinal villi is dependent on the indigenous microbes. This has been demonstrated in studies using germ-free mice and its subsequent colonization by *B. thetaiotamicron* by Jeffrey Gordon's group [17]. This signifies the importance of the gut microbes in the development of the structure and morphology of the gut (Figure 2).

### 2.2. Metabolic Functions

The gut bacteria are known to produce a large number of vitamins like the B group of vitamins, synthesize amino acids, and carry out biotransformation of the bile. Biotransformation of bile by microbial enzymes is important for the metabolism of glucose and cholesterol [18]. Importantly, the microbiome provides the much needed biochemical pathways for the fermentation of nondigestible substrates like fibers and endogenous mucus. Fermentation or metabolism of these nondigestible substrates leads to the growth of these microbes and the production of short chain fatty acids and gases [19]. The major short-chain fatty acids produced are acetate, propionate, and butyrate. Other bacterial end products include lactate, ethanol, succinate, formate, valerate, caproate, isobutyrate, 2-methyl-butyrate, and isovalerate. Bacterial fermentation takes place in the cecum and colon, where the short-chain fatty acids are absorbed, stimulating the absorption of salts and water. These short-chain fatty acids have a protective effect on the intestinal epithelium [19]. The colonic bacteria prefer butyrate as the sole source of energy, and most of it is completely metabolized. The principal short chain fatty acid produced in the colon is acetate, and it serves as a substrate for biosynthesis of cholesterol. Thus the gut microbiota performs various metabolic acitivities which are essential for the host's metabolism (Figure 2).

### 2.3. Protective Functions

Many of the commensal organisms produce antimicrobial compounds and compete for nutrients and sites of attachment in the gut lining, thereby preventing colonization by pathogens. This helps reduce the production of lipopolysaccharides and peptidoglycans which can all be detrimental to the host [20]. The development of the immune system is also governed by the nature of the indigenous microflora [21]. Germ free animals have fewer dendritic cells, and evidence shows that bacterial systems have a role to play in development of B cells [22, 23]. The development of regulatory T cells, T helper type 1 and 2 cells, and T helper 17 cells is also dependent on the signals given by the intestinal bacteria [24–26]. Short-chain fatty acids, such as butyrate, have been shown to inhibit NF-kB in patients with ulcerative colitis thus exerting immunomodulatory effects [27, 28]. 

These concepts illustrate a dynamic relationship between the immune system and the microbiota. The intestinal mucosa averts threats by signaling to the innate immune system through toll-like receptors. These recognize and bind to specific microbial macromolecules, like lipopolysaccharide, flagellin, peptidoglycan, and N-formylated peptides. In the intestinal mucosa, the activation of toll-like receptors initiates nuclear factor-kB pathways, mitogen-activated protein kinase, and caspase-dependent signaling cascades. These lead to the production and release of protective peptides, cytokines, chemokines, and phagocytes. The result can be a protective response to commensal bacteria, an inflammatory response to pathogenic organisms, or a trigger of apoptosis. Therefore, commensal bacteria of the gastrointestinal tract play active roles in the development and homeostasis of the immune system, as shown in Figure 2.

## 3. Dysbiosis and Modulating of the Gut Microbiota

**Figure 3:**
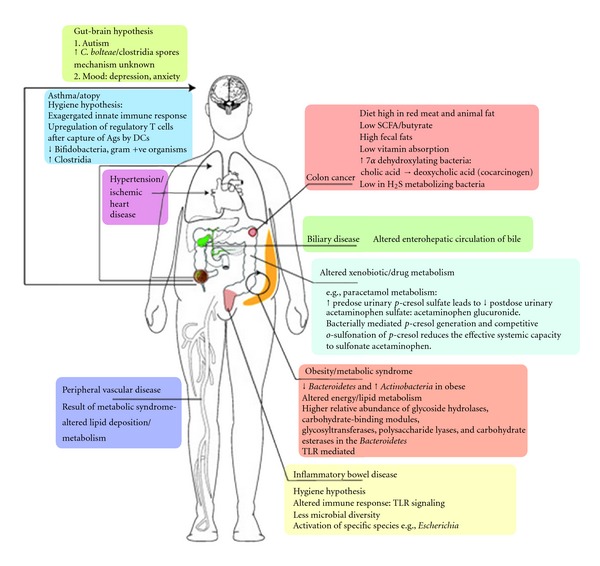
Diseases influenced by gut microbial metabolism. The variety of systemic diseases that are directly influenced by gut microbial metabolism and its influence on other mammalian pathways, such as the innate immune system, are shown. Specifically highlighted are the metabolic pathways involved in drug metabolism and obesity that are directly influenced by the gut microbial content. Ags, antigens; *C. bolteae*, *Clostridium bolteae*; DCs; dendritic cells; SCFA, short-chain fatty acid; TLR, toll-like receptor. Kinross et al. Genome Medicine 2011 3:14.

Normal physiology of the host depends on the signals given by the intestinal microbes. The intestinal lumen consisting of gastric acid, digestive enzymes, and IgA constitutes the first line of defense and is lethal to invading and ingested pathogenic bacteria. The indigenous microbes degrade intraluminal antigens and inhibit the pathogenic microbes from adherence and colonization. They also are necessary for the induction of regulatory T cells [29]. Any changes to this microbial ecosystem could cause an imbalance or dysregulation of the microbiota (dysbiosis) often associated with various disease states ranging from the most common IBD [30, 31] and IBS [32] to the more unexpected activation of chronic human immunodeficiency virus (HIV) infection [33] and generation of atopy [34–36] (Figure 3).

It is therefore important to reestablish the bacterial homeostasis which may have been disturbed by any or several factors. One of the ways to favorably alter the intestinal microbiota is through the use of prebiotics, probiotics, and synbiotics (a combination of both prebiotics and probiotics given together). These agents can favorably influence microbial interactions with the immune system and gut epithelium. 

A *prebiotic* is a selectively fermented ingredient that results in specific changes in the composition and/or activity of the gastrointestinal microbiota, thus conferring health benefit(s) upon the host. Prebiotics are generally oligomers made up of 4 to 10 monomeric hexose units.

*Probiotics*, according to the currently adopted definition by FAO/WHO [37, 38], are “Live microorganisms which when administered in adequate amounts confer a health benefit on the host." The International Scientific Association for Probiotics and Prebiotics (ISAPP with Glenn Gibson, Todd Klaenhammer, and Mary Ellen Sanders on its board of directors) and the International Probiotic Association (IPA, an association of over 150 probiotic business organizations manufacturing and distributing probiotics) are two groups which are working with these beneficial microbes.

*Synbiotics *is a combination of probiotics and prebiotics administered together.

Common, well-known beneficial bacteria which have a long-standing association with health include lactic acid producing genera such as the *Bifidobacteria* or *Lactobacilli*. These bacteria can be introduced into the gut and/or encouraged to multiply either through ingestion by the individual of appropriate probiotic strains or through the provision of prebiotic growth substrates also known as soluble fibers.

That probiotics and prebiotics are becoming increasingly popular is evidenced by rapidly expanding research support and an ever widening choice of products. Probiotics and prebiotics are available commercially in many forms, including foods, dietary supplements, and clinical therapeutics with oral or non-oral delivery.

To be a candidate for commercialization, a probiotic must retain its properties during large-scale industrial preparation. Naturally, it should also remain viable and stable during storage and use. For most applications, the probiotic should be able to survive in the intestinal ecosystem and the host animal should gain beneficially from its presence. Clearly, the organisms used should be “generally regarded as safe”-GRAS as per USFDA regulations or well documented in the literature.

Prebiotics must provide selective stimulation of the growth or activity of beneficial native bacteria. Since prebiotics are non-viable, stability is not a concern, but safe consumption levels must be established. A detailed guideline for probiotics and prebiotics has been published by the World Gastroenterology Organization [39].

## 4. Clinical Applications of Various Probiotics, Prebiotics and Synbiotics

### 4.1. Gut Microbiota and Obesity

The metabolic equilibrium of the host is maintained by the gut microbes [40, 41]. One study in adult population with type 2 diabetes [42] has shown that their gut microbiota differs from that of non-diabetic adults, and that health may potentially improve when the gut microflora is modified by the administration of probiotics and prebiotics. In spite of these findings, and the relationship between diabetes and abdominal fat, few studies have been aimed at finding correlations between the composition of the microbiota and the occurrence of inflammation and metabolic alterations in individuals with obesity [42, 43]. A study in patients with diabetes mellitus showed that these individuals had a lower number of *Faecalibacterium prausnitzii* and an increase in with inflammatory markers [43]. Obesity was found to be associated with large changes in the abundances of different bacteria from different taxa [44]. 

The *Bifidobacteria* population (and most other organisms in the group of Firmicutes) is slightly lower in individuals with obesity than in lean people [45]. A similar finding was reported in patients with type 2 diabetes mellitus in comparison with nondiabetic patients [46]. These findings suggest that Bifidobacteria may play a part in the development of obesity and its related comorbidities. When prebiotics like inulin-type fructans were fed to mice, these were used as energy substrates by bacteria [47–49]. The number of Bifidobacteria increased significantly, and there was an inverse correlation with the levels of lipopolysaccharide, glucose tolerance and development of fat mass [47, 48]. Moreover the prebiotic approach prevented the overexpression of several host genes that are related to adiposity and inflammation.

Studies have been carried out using probiotics to promote specific changes in the gut microbiota. Angiopoetin-related protein 4 (Angptl4), a lipoprotein lipase inhibitor which inhibits the uptake of fatty acids from circulating triglyceride-rich lipoproteins in white adipose and muscle tissues was found to be increased in mice fed with a high fat diet supplemented with *L. paracasei* [50]. Obese individuals when administered with *Lactobacillus acidophilus* NCFM and *Lactobacillus gasseri* SBT2055 showed a decrease in fat mass and the risk of type 2 diabetes mellitus and insulin resistance [51, 52]. In the active group which consumed *L. gasseri*, abdominal, visceral, and subcutaneous fat areas decreased significantly. Body weight also decreased significantly. In the *L. acidophilus* NCFM study the insulin sensitivity was preserved, but there was no effect on the systemic inflammatory response. Clinical trials using prebiotics like arabinoxylan [53–55] and inulin-type fructans [56–58] have shown positive results in diabetic, overweight, and obese populations. A review article [59] discusses the tight relationship which exists between mammalian gut composition and functions and the host metabolism using modern molecular techniques. Gut microbes can affect host metabolism and energy storage and thus predisposition to obesity and diabetes. 

### 4.2. Allergy and Atopic Diseases of Children

Atopic diseases arise from aberrant immune responses to environmental allergens leading to allergic inflammation [60]. The allergic responses are mediated by the Th2 cells which produce interleukins-4, -5, -9, and -13. Genetics play a strong role, and genes-encoding proteins which are involved in the pathogenesis of allergic inflammation have been identified [61, 62]. Atopic dermatitis (AD) a common allergic skin disease is widely prevalent in children from US and Western Europe [63]. Children suffering from AD have higher number of *S. aureus* and *Clostridium* in their colon and lower number of *Enterococcus*, *Bifidobacterium,* and *Bacteroides* [64, 65]. With the increasing recognition of the importance of healthy intestinal microbiota, there has been a substantial effort to assess the potential role of probiotics in the prevention and/or treatment of allergic diseases in human clinical trials. When *Lactobacillus* GG was administered to high risk infants, there was a 50% reduction in observed atopic eczema [66]. In another study in Finland when children were given a whey formula with *L. rhamnosus* or *B. animalis* ssp. lactis for 2 months, the skin condition improved [67]. Similar curative results were obtained *L. rhamnosus* plus *L. reuteri* preparations [68].

In another study, *Lactobacillus fermentum* reduced symptoms of atopic dermatitis in infants with moderate-to-severe disease [69]. Supplementation with *L. rhamnosus* HN001 in pregnant women and their newborn infants substantially reduced the cumulative prevalence of eczema in infants [70]. A probiotic cocktail of *Bifidobacterium bifidum*, *Bifidobacterium lactis*, and *Lactococcus lactis* was able to significantly reduce eczema in high-risk infants for a minimum of 2 years provided that the probiotic was administered to the infant within 3 months of birth [71]. A double blind, randomized, and placebo-controlled intervention in children with atopic dermatitis (AD) using Danisco's probiotic strain *Bifidobacterium animalis* subsp lactis. Bi-07 showed that there was a significant reduction in the severity of AD with an improved ration of IFN-*γ* and IL-10 [72]. Other studies also indicate that the consumption of dietary supplements or foods containing probiotics can stabilize the intestinal barrier function and decrease gastrointestinal inflammation in children with AD [73].

### 4.3. Hepatic Encephalopathy

Hepatic encephalopathy is a dreaded liver disease. Minimal encephalopathy is a condition of chronic liver disease with no clinical symptoms of brain dysfunction. The exact pathogenesis of hepatic encephalopathy is still unknown, and the basis for it is still not completely understood [74]. However it is widely agreed that gut-derived-nitrogenous substances and, specifically, ammonia derived primarily from enteric bacteria play a central role. Use of probiotics for MHE has been rationalized based on various modes of action like decreasing bacterial urease activity, decreasing intestinal permeability, decreasing inflammation, decreasing uptake of other toxins, and other modes of action. Use of probiotics has been demonstrated to result in reduced concentrations of many bacteria [75], particularly gram-negative bacteria which produce urease. They have also been shown to improve intestinal permeability in experimental human models [76]. A rat model of hepatic failure has shown that certain bacteria can produce a ligand for the benzodiazepine receptor that may contribute to the encephalopathy [77]. When patients with minimal hepatic encephalopathy were given *Bifidobacterium longum* with fructooligosaccharide for 9 weeks, their cognitive functions were seen to improve [78].

Endotoxemia causes inflammation leading to cirrhosis of the liver. When fecal flora of cirrhosis patients was analysed, there was a substantial reduction in the levels of *Bifidobacteria* [79]. Minimal hepatic encephalopathy (MHE) is a complication of cirrhosis during which accumulation of neurotoxic substances in the bloodstream produces neurological manifestations. When MHE patients were given a synbiotic preparation of probiotics and prebiotics, the MHE was reversed in 50% of the patients, and this effect was accompanied by a significant increase in *Lactobacilli* [80].

A recent review on the role of probiotics for hepatic encephalopathy concludes the need for further random trials before probiotics can be endorsed for hepatic encephalopathy [81].

### 4.4. Hypocholesterolaemic and Cardioprotective Effects

Hypercholesterolemia, or elevated level of total cholesterol in the bloodstream, is the result of high levels of low-density lipoprotein (LDL) as compared to high-density lipoprotein (HDL) cholesterol. Many *Lactobacilli*, being the natural inhabitants of the intestine, possess bile-salt hydrolase activity. This property has been used for developing probiotic formulations to combat hypercholesterolemia. 

Many animal models have been used to evaluate the effects of probiotics and prebiotics on serum cholesterol levels in many studies. When Abd El-Gawad used buffalo milk-yogurt fortified with *B. longum* in male albino rats for 35 days, total cholesterol was reduced by 50%, LDL-cholesterol by 56%, and triglycerides by 51% in comparison to the control [82]. When *L. plantarum* PH04 was evaluated for its cholesterol lowering effects in rats, the total serum cholesterol and triglyceride levels showed a significant reduction as compared to the control [83]. In hypercholesterolemic male rats, fed over a four-week period with rice bran fermented with *L. acidophilus*, a significantly improved lipid profile was obtained when compared to the control [84]. 

Studies with humans have shown similar results. In a 10-week randomized, double-blind, placebo-controlled, and crossover study with *L. acidophilus* L1 milk, there was a significant reduction in serum cholesterol compared to the placebo group [85]. Xiao et al. [86] evaluated the effects of a low-fat yogurt containing *B. longum* BL1 in a randomized, single blind, placebo-controlled and parallel study involving thirty-two patients. At the end of 4 weeks, the patients showed a significant decline in total serum cholesterol, LDL-cholesterol and triglycerides. There was also a 14.5% increase in HDL cholesterol when compared to the control.

Some studies with prebiotics have also been carried out. A randomized, double blind, and crossover study in hamsters used inulin as a prebiotic. The result was a 29% decrease in total cholesterol and a 63% decrease in triglycerides [87]. A study with 40 male Sprague-Dawley rats showed a 27% reduction in triglycerides when xylooligosaccharide was used as a prebiotic [88]. Causey et al. [89] conducted a randomized, double-blind, and crossover study in twelve hypercholesterolemic men in order to assess the effects of inulin in blood cholesterol. Twenty grams of inulin were given daily. There was a significant reduction of serum triglycerides at the end of the 3-week study.

Synbiotics have also been evaluated for their hypocholesterolemic effects. Twenty-four hypercholesterolemic male pigs were fed with a synbiotic formulation of *L. acidophilus* ATCC 4962, fructooligosaccharides, mannitol, and inulin. Positive results were obtained at 8 weeks. Total plasma triacylglycerol, total cholesterol, and LDL levels decreased [90]. Kießling et al. [91] evaluated a synbiotic yogurt containing *L. acidophilus* 145, *B. longum* 913, and oligofructose in a randomized, placebo-controlled, and crossover study involving twenty-nine women. The HDL cholesterol increased. In yet another study, Schaafsma et al. [92] saw a significant decline in total cholesterol and LDL cholesterol in thirty volunteers who were fed synbiotic milk containing *L. acidophilus* and fructooligosaccharides.

Many studies have convincingly demonstrated cholesterol-lowering effects of probiotics in both animals and humans. However some controversial results have also been observed. Double blind, randomized, and crossover studies using *L. rhamnosus* LC705 [93], parallel design studies using *L. fermentum* [94], and crossover studies using *L. acidophilus* [95] showed no change on serum lipids, triglycerides, or cholesterol. Similar controversies were also raised from studies evaluating the hypocholesterolemic properties of prebiotics. When a diet with flaxseed at 1.3 g/100 g was given in a controlled, double-blind, and crossover study, there was no significant change in blood lipids [96]. Another study, using 20 gm/day of fructooligosaccharides for a period of 4 weeks in type 2 diabetes patients showed no effect on glucose and lipid metabolism [57]. Similar results were obtained on lipid modulation in a study with 18 g/day of inulin [97]. One study using a synbiotic preparation of *Lactobacillus acidophilus*, *Bifidobacterium longum*, and fructo-oligosaccharides in women over a 2-month period, also showed no changes in plasma concentration of total cholesterol, HDL cholesterol, LDL cholesterol, and triglyceride [98].

### 4.5. Cancer Prevention

As early as 1995, in a controlled, double blind study, with 138 patients a *L. casei* Shirota preparation was shown to have a preventive effect on the recurrence rate of superficial bladder cancer after surgery [99]. In different animal models (rats and mice) fed with inulin and/or oligofructose did reduce the genotoxicity of fecal water [100]. It also decreased the number of chemically induced precancerous lesions [101, 102] and stimulated defense functions. An increased level of IL-10 and of NK-cell activity was also observed [103]. In the long term, the tumor incidence in the large intestine [104] and in other organs (breast cancer in rats and mice, metastases in the lung [105]) was lowered by adding from 5 to 15% inulin or oligofructose to the diet. This effect was even more pronounced when a combination of prebiotics and probiotics was given [106]. Xylooligosaccharide was shown to reduce the number of aberrant crypt foci in the colon of 1, 2-dimethylhydrazine-treated male Sprague-Dawley rats [88].

Some of the probiotic strains which have been/are being used for different cancers, along with their references, are summarized in Table 1.

### 4.6. Probiotics and Renal Health

It has been demonstrated that gut microflora can affect the concentrations of uremic toxins in animals. Prakash and Chang were able to continuously reduce blood urea nitrogen in azotemic rats by oral administration of microencapsulated genetically engineered live cells containing living urease-producing *E. coli* DH5 [115]. Based on this concept, Ranganathan et al. [116] carried out rat studies using 5/6th nephrectomised animals fed with a probiotic cocktail of *Lactobacilli*, *Bifidobacteria*, and *S. thermophilus*. Results showed a significantly prolonged life span for the uremic rats, in addition to reduced blood urea-nitrogen (BUN) levels. Studies were subsequently carried out in 5/6th nephrectomised Gottingen mini pigs [117]. Here also there was a reduction in BUN and creatinine levels, indicating that the probiotic supplementation prevented the accumulation of these toxins in the blood. These results were further evaluated clinically by Richard Palmquist [118] in feline azotemia. Studies in 7 cats showed statistically reduced levels in BUN and creatinine levels and demonstrated significantly improved quality of life (QOL).The product is currently marketed for cats and dogs with moderate-to-severe kidney failure (as “Azodyl” by Vetoquinol SA with worldwide veterinary product sales (http://www.vetoquinol.com/)).

In human studies, Simenhoff et al. demonstrated that hemodialysis patients who were fed *L. acidophilus* NCFM had significantly lower blood dimethylamine and nitrodimethylamine levels [119, 120]. Simenhoff was the first researcher to demonstrate the growth of pathogenic bacteria which is referred to as “Small Bowel Bacterial Overgrowth” (SBBO). The NCFM strain is well known, and the genome has been sequenced by Todd Klaenhammer's group [121]. Subsequent to the success of the formulation for cats and dogs described above, a similar formulation for humans was evaluated clinically in a 6-month randomized, double-blind, placebocontrolled, and crossover trial in CKD stage 3 and 4 patients in four countries [122, 123]. 46 patients were studied in this trial. BUN levels decreased in 29 patients (*P* < 0.05), creatinine levels decreased in 20 patients (no statistical significance), and uric acid levels decreased in 15 patients (no statistical significance). Almost all subjects reported having experienced a substantial perceived improvement in their quality of life (*P* < 0.05). This product is also currently marketed by Kibow Biotech, Inc. with the brand name “Renadyl” (http://www.renadyl.com/).

## 5. Future Emerging Areas for Probiotic Research

### 5.1. Myocardial Infarction

Intestinal microbiota has also been shown to promote cardiovascular disease, specifically atherosclerosis, by their catabolism of choline [124, 125]. There is a preliminary evidence that the use of probiotic *Lactobacilli* and its metabolic byproducts potentially confer benefits to the heart, including prevention and therapy of various ischemic heart syndromes [126] and reduction of serum cholesterol [127]. When *L. plantarum* 299v was supplemented to the diet of smokers, the serum levels of leptin and fibrinogen and LDL-cholesterol, the risk factors for cardiovascular disease, were also reduced [128]. When rats were fed fruit juice containing *L. plantarum* 299v and *B. lactis* Bi-07, the study results showed that this probiotic supplementation decreased circulating leptin levels and reduced myocardial infarction to the same extent as with the use of vancomycin [129].

### 5.2. Gut-Brain and Behavior

Exactly how the microbiota influence brain behavior is still unknown but an explanation could involve immune-mediated neural or humeral mechanisms. Dr. Gershon, an expert in neurogastroenterology and the author of “The Second Brain” [130], evokes the possible existence of an enteric nervous system, the second brain, which would consist of sheaths of neurons embedded in the gut wall. Dendritic cells in the GI tract have processes that enable them to breach the epithelial layer and interact with commensal bacteria to induce the production of immunoglobulin A by B lymphocytes and plasma cells [131]. The secreted immunoglobulin A prevents the microbiota from penetrating the epithelium. Dendritic cells are in close proximity to nerves in the GI tract [132], and its function is modulated by the sensory neuropeptide calcitonin-gene-related peptide [133]. This might signal the presence of commensal bacteria to the brain by the vagus nerve [132]. The vagus nerve has an important role in signalling from the GI tract to the brain and can be stimulated by bacterial products such as endotoxins or inflammatory cytokines such as interleukin-1*β* and tumor necrosis factor *α* [134]. The vagal response also leads to suppression of proinflammatory cytokine release from intestinal macrophages [134, 135].

Recent studies have demonstrated the ability of probiotics to influence psychological states. Mark Lyte talks of “Microbial Endocrinology,” a new interdisciplinary field, which addresses the ability of probiotics to both synthesize and respond to neuroactive compounds as a mechanism by which biological processes of the host, both physiological and neurological, may be influenced [136–139]. Many probiotic bacteria produce neurochemicals which are identical to those produced by mammalian systems (Table 2) [140]. The presence of catecholamine biosynthetic pathways in bacteria [141] indicates the possibility that cell-to-cell signalling in vertebrates may be due to late horizontal gene transfer from bacteria [141]. Mark Lyte hypothesizes about the use of probiotics as delivery vehicles for neuroactive compounds based on this new field of microbial endocrinology [142].

In an experiment with rats which were subjected to a forced swim test to test their behavior, feeding of *Bifidobacterium infantis* resulted in neurochemical alterations and an increase in proinflammatory response that suggested a potential antidepressant capability for the administered probiotic [143]. When mice were chronically infected with *H. Pylori*, they too showed an evidence of behavioral changes. There was an alteration in the feeding behavior when compared to healthy controls. The infected mice showed early satiety. There were also elevated levels of TNF-*α* in the CNS [144]. Another study in rats subjected to a water avoidance stress test showed that when they were fed with a probiotic combination of *L. rhamnosus* and *L. helveticus*, there was a reduction in the chronic psychological stress [145]. Similar results were obtained by Eutamene et al. [146] in rats fed with *L. paracasei*. There was an improvement in stress-induced visceral pain. 

Administration of a probiotic formulation consisting of *Lactobacillus helveticus* RO052 and *B. longum* RO175A significantly attenuated psychological distress in human volunteers and reduced anxiety-induced behavior in a rat model [147]. In another study, a combination of probiotic cultures and multivitamin/minerals has been shown to improve depressive symptoms in a group of fatigued adults under stress [148]. Chronic fatigue syndrome patients treated with *Lactobacillus casei* strain “Shirota” for two months showed a significant rise in both *Lactobacillus* and *Bifidobacteria* resulting in improved patient outcome [149].

### 5.3. Familial Mediterranean Fever

The first genetic disease to be linked to changes in healthy gut flora is Familial Mediterranean fever (FMF). FMF provides evidence that host genotype can dictate the establishment and composition of the intestinal flora. In FMF, the gene for pyrin an important regulator of innate immunity is mutated. This leads to an autoinflammatory disorder called FMF. Khachatryan studied 19 FMF patients. They found significant reduction in the total number of bacteria, and ratios of the types of bacteria of the genus Bacteroidetes, Firmicutes and Proteobacteria [150]. In a subsequent study, 15 gut bacteria belonging to *Bacteroides*, *Lactobacillus*, *Escherichia*, *Enterococcus*, and *Parabacteroides* were chosen. The levels of systemic antibody response IgG and IgA towards these were studied in healthy and FMF disease populations using ELISA. The total IgG titer in FMS patient increased by 35 percent compared to the control, suggesting that the functionality of pyrin affects the ability of commensals to breach the gut barrier, resulting in characteristically high systemic reactivity towards these bacteria [151].

### 5.4. Autism

Very little is known about the underlying etiology of autism. Extensive antibiotic use is commonly associated with late-onset autism (18–24 mo of age), causing some to hypothesize that disruptions in the normal microbiota may allow colonization by autism-triggering microorganism(s), or promote the overgrowth of neurotoxin-producing bacteria like *Clostridium tetani* [152]. Finegold et al. [153] suggested a number of mechanisms whereby the gut microbiota could be responsible for the debilitation of regressive autism including neurotoxin production by a subset of abnormal flora, autoantibody production that results in the attack on neuron-associated proteins, or microbial production of toxic metabolites that have neurological effects [153].

## 6. Drug Interactions of Probiotics

 Drugs are known to interfere with administered probiotics [10]. Interactions between probiotics and warfarin are known [154, 155]. There is also some potential for interaction with warfarin (Coumadin); for example, Bifolac is a probiotic that is used for normalisation of gut flora, prophylactic, or temporary gastrointestinal disorders. The drug contains two bacterial strains: *Lactobacillus rhamnosus *and *Bifidobacterium longum*. The risk of potential drug interactions with Bifolac has not been studied [154, 155].

Since probiotics contain live microorganisms, concurrent administration of antibiotics could kill a large number of the organisms, reducing the efficacy of the *Lactobacillus *and *Bifidobacterium *species. Patients should be instructed to separate the administration of antibiotics from these bacteria-derived probiotics by at least two hours [156, 157]. Similarly, *S. boulardii *might interact with antifungals, reducing the efficacy of this probiotic [158]. According to the manufacturer, Florastor, which contains *S. boulardii*, should not be taken with any oral systemic antifungal products [159]. Probiotics should also be used cautiously in patients taking immunosuppressants, such as cyclosporine, tacrolimus, azathioprine, and chemotherapeutic agents, since probiotics could cause an infection or pathogenic colonisation in immunocompromised patients [156–158].

Warfarin is known as a vitamin K antagonist and acts by blocking the intracellular activation of vitamin K. Intestinal bacteria produce a significant proportion of the vitamin K absorbed in the intestine locally, while antibiotics causing the disruption of the intestinal flora has been associated with symptomatic K vitamin deficiency and severe hemorrhage [160, 161]. It is therefore conceivable that administration of bacteria that alter the local production of vitamin K could affect the sensitivity to warfarin and other vitamin K antagonists. 

Animal studies indicate that *B. longum* lacks the ability to synthesize vitamin C [162], and* in vitro* studies using the bacterial strain showed a decrease in vitamin K levels of the culture medium. Vitamin C acts as a growth factor for many bacterial strains, and since *B. longum* only requires low levels of the vitamin for its growth, we can interpret its ability to lower vitamin K concentrations in the surrounding area as a means of competing with other strains that depend on high vitamin K levels [163].

While this may result in a theoretical risk of interactions with warfarin based on the proposed mechanism, clinical significance has yet to be shown. No information on *L. rhamnosus'* possible role in the production and metabolism of vitamin K has been found. In view of the theoretical interaction potential, careful international normalized ratio (INR) monitoring of warfarin-treated patients starting Bifolac treatment is recommended [164].

Other than the influence of dietary vitamin K intake, there is essentially no experimental or clinical evidence that any particular food or nutrient will interact with warfarin through modulation of CYP2C9 activity [164–167]. Further controlled studies should be conducted to determine if actual interaction potentials exist [10, 164, 165].

## 7. Safety of Administration of Probiotics

Consumers are increasingly using probiotics for their various health benefits. In healthy individuals probiotics are safe to be used. Clinical evidence for their efficacy is strong in case of antibiotic-associated diarrhea management [168]. However there are areas of uncertainty. Caution has to be exercised with certain patient groups like premature neonates or immune deficiency. Paucity of information regarding the mechanisms through which probiotics act, appropriate administrative regimes, and probiotic interactions necessitate further investigations in these areas. Properties of probiotics are strain specific. Hence confirmation studies need to be performed, and effects cannot be generalized. A detailed assessment by NIH on the safety aspects has been published [169]. The conclusion which has emerged necessitates the need for systematic reporting of adverse events and better documentation of interventions.

## 8. Human Microbiome and Human Health

The Human Microbiome project launched in 2008 by the National Institute of Health (NIH) aims to understand if changes in the human microbiome are associated with human health or disease. These studies have revealed that even healthy individuals differ remarkably in the microbes that occupy different body sites or habitats. Different groups are studying the microbiology of five body sites oral, skin, nasal, gut and urogenital. A study with 242 healthy adults analysed 4,788 specimens from different body habitats [170]. Analysis showed that no taxa were observed to be universally present among all body habitats and individuals studied. Inter-individual variation in the microbiome was specific and personalized. Even within the same species there were strain level genomic variations. Jeffrey Gordon's group studied a wider and diverse population from different geographic, socioeconomic, and cultural settings. The study comprised of healthy adults and children including mono- and dizygotic twins from the Amazons of Venezuela, rural Malawi, and the US metropolitan areas [171]. A total of 531 individuals were studied. Single fecal sample from each individual was analysed. 16S rRNA genes present in the feces were analysed to define the phylogenetic types. The findings are interesting and notable. Interpersonal variation was significantly greater among children. There were significant differences in the phylogenetic composition of fecal microbiota between individuals living in the different countries. The fecal microbiota of US adults was the least diverse. An important observation was the degree of similarity among family members regarding the microbial community structure across the three populations studied. Another group has studied the carbohydrate metabolizing enzymes in the five body sites [172]. Carbohydrate metabolizing enzymes are specific to bacterial taxa, and different body sites are inhabited by different bacterial communities. Despite this, complex carbohydrate cleaving enzyme (CAZymes) profiles were found to be very similar within a body site. This suggests quote “the carbohydrate composition of each body site has a profound influence and probably constitutes one of the major driving forces that shapes the community composition and therefore the CAZyme profile of the local microbial communities, which in turn reflects the microbiome fitness to a body site.” Yet another study using new statistical analysis showed that highest diversity was present in stools. Oral and skin habitats had variable diversity patterns while vaginal habitats were the least diverse [173].

Such varied studies would enable us to understand the complex relationship between human health and the human microbiome and might pave the way to better management of health using beneficial microbes for disease prevention/management.

## 9. Concluding Remarks

As the gut microbiota appears to contribute to nearly every aspect of the host's growth and development, it is not surprising that a tremendous array of diseases and dysfunctions have been associated with an imbalance in either composition, numbers, or habitat of the gut microbiota.

Probiotics, prebiotics, and their combinations have been found to be clinically effective for a large number of gut based disorders like IBD, digestion, travelers diarrhea, and for improving/helping to maintain general health. Emerging areas of research have shown promise in cancer, brain, kidney, and obesity. It remains to be seen whether probiotics and prebiotics can be effective in combating diseases like autism, pancreatitis, fibromyalgia, etc.,) where dysbiosis has been observed. The future is going to be challenging but promising, since tools for probiotic research are now available. Much work has already been accomplished to help us understand probiotics and the manner in which they function. Therefore the field of probiotics, prebiotics and synbiotics may potentially open a new branch of science, paving a new way for personalized medicine, and maybe even future biotherapeutics. 

## Figures and Tables

**Table 1:** Various probiotic strains and their applications in different types of cancer and side effects associated with cancer.

Sr No	Strain	Indication	Results	References
(1)	*L. rhamnosus* GG +* B. lactis* Bb12 (10B each with inulin)	Polypectomized and colon cancer patients	Increased *L. rhamnosus* and *B lactis* in feces, reduction in *C perfringens*, prevents increased secretion of IL-2 in polypectomized patients, increased production of interferon-*γ* in cancer patients.	[107]
(2)	*L. casei* Shirota 30B daily	Recurrence of superficial bladder cancer	Significant reduction in primary multiple and recurrent single tumors	[108]
(3)	*L. casei *Shirota	Preventive effect on bladder cancer	Significant reduction in risk of bladder cancer	[109]
(4)	*L. casei *LC9018	Cervical cancer	Reduced immunity against tumor induction	[110]
(5)	*L. plantarum* CGMCC No 1258, *L. acidophilus* LA-11, *B. longum* BL-88. Daily dose of 2.6∗10^14^CFU	Barrier function and post-operative infectious complications in Colorectal cancer surgery	Improvement in the integrity of gut mucosal barrier and decrease in infectious complications	[111]
(6)	*L. acidophilus *and *B. bifidum* 1B CFU each	Diarrhea during radiotherapy in cervical cancer	Reduction in incidence of diarrhea and better stool consistency.	[112]
(7)	VSL#3	Radiation induced diarrhea	Less diarrhea, improvement in daily bowel movements	[113]
(8)	*L. rhamnosus* GG 10 to 20B daily for 24 weeks	Diarrhea related to chemotherapy of colorectal cancer	Patients had less grade 4 or 4 diarrhea, less abdominal discomfort, needed less hospital care and had fewer chemo dose reduction due to bowel toxicity.	[114]

**Table 2:** Neurochemicals isolated from various microbes (as from [140]).

Genus	Neurochemical
*Lactobacillus and Bifidobacterium*	GABA
*Escherichia, Bacillus, and Saccharomyces*	Norepinephrine
*Candida, Streptococcus, Escherichia, and Enterococcus*	Serotonin
*Bacillus and Serratia*	Dopamine
*Lactobacillus*	Acetylcholine
